# Smaller Is Better: Drift in Gaze Measurements due to Pupil Dynamics

**DOI:** 10.1371/journal.pone.0111197

**Published:** 2014-10-22

**Authors:** Jan Drewes, Weina Zhu, Yingzhou Hu, Xintian Hu

**Affiliations:** 1 Centre for Vision Research, York University, Toronto, Canada; 2 Center for Mind/Brain Sciences, Trento University, Rovereto, Italy; 3 School of Information Science, Yunnan University, Kunming, China; 4 Kunming Institute of Zoology, Chinese Academy of Sciences, Kunming, China; Tokyo Metropolitan Institute of Medical Science, Japan

## Abstract

Camera-based eye trackers are the mainstay of eye movement research and countless practical applications of eye tracking. Recently, a significant impact of changes in pupil size on gaze position as measured by camera-based eye trackers has been reported. In an attempt to improve the understanding of the magnitude and population-wise distribution of the pupil-size dependent shift in reported gaze position, we present the first collection of binocular pupil drift measurements recorded from 39 subjects. The pupil-size dependent shift varied greatly between subjects (from 0.3 to 5.2 deg of deviation, mean 2.6 deg), but also between the eyes of individual subjects (0.1 to 3.0 deg difference, mean difference 1.0 deg). We observed a wide range of drift direction, mostly downward and nasal. We demonstrate two methods to partially compensate the pupil-based shift using separate calibrations in pupil-constricted and pupil-dilated conditions, and evaluate an improved method of compensation based on individual look-up-tables, achieving up to 74% of compensation.

## Introduction

In current research involving eye tracking, video-based systems are almost omnipresent. They are being offered in a wide variety of models and technical specifications, varying in design (head mounted, tower mounted, display-mounted etc.) but also temporal and spatial resolution and accuracy. Virtually all video-based systems rely on a mechanism that tracks the pupil in the recorded video image, and uses the computed center of the pupil as a primary indicator of gaze direction. This technique generally assumes a fixed alignment between the “true” visual axis of the eye and the computed center of the pupil. The motion of the pupil should therefore accurately represent the motion of the eyeball, and in consequence is assumed to indicate the direction of gaze. However, recently it has been reported that changes in pupil size are highly correlated with apparent changes in gaze direction, even under conditions where gaze direction would not be expected to change [1]. A primary reason for changes in pupil size is a change in luminance inside the visual field, possibly resulting in high amplitudes of pupil dilation or constriction, at latencies that are highly relevant in eye movement research (130-500ms, [2], [3]. However, even when luminance is constantly controlled to the highest standards, pupil dilation and constriction may still occur due to a number of other factors, which can be task-related such as general mental workload [4], [5], especially in human-computer interaction [6], or more specifically target detection [7], [8], but also task unrelated, such as independent emotional processes, the first systematic report of which was probably [9]. Even scene content [10] can influence pupil size. Ultimately, from the perspective of the eyetracking system, the reasons for changes in pupil size are not relevant, as all changes in pupil size are effected through the same motor system.

To date only two studies have analyzed this phenomenon, reporting amplitudes of up to 1.2 degrees based on 5 subjects [1] and up to 2.4 degrees based on 2 subjects [11]. The latter study provided ground truth by means of simultaneously measuring with two different eye tracking devices, proving the drift to be a measurement artifact rather than an actual eye movement. However, the small number of subjects in both studies is not sufficient to provide a useful overview of the average magnitude of the pupil-size induced gaze measurement drift, and it is unclear if other drift characteristics such as direction both across individual subjects and between the eyes of the same subject are completely random or if there exists a common trend that may be found in the general population.

To shed more light on the phenomenon of pupil-size induced drift in measured gaze position, we present data recorded from a larger population of subjects, and we propose several approaches to compensate the observed measurement drift.

## Methods

### Experimental Setups

Data was collected at two different locations. 21 subjects were collected at the Kunming Institute of Zoology CAS, Kunming, China(1). 18 more subjects were collected at the Center for Mind/Brain Sciences, Trento University, Rovereto, Italy(2). Experiments were conducted according to the principles expressed in the declaration of Helsinki and were approved by the ethics committees of the Kunming Institute of Zoology as well as Trento University. All subjects provided informed written consent. Stimuli were displayed using either a Samsung 22″ LCD display(1) or a ViewSonic 20″ CRT display(2). Viewing distance was 60 cm with the head supported by a combined chin/forehead rest to maintain a stable head position within the field of view of the eye tracker. To allow for a sufficiently dark environment, no external lighting other than the stimulus display was used. Both labs used Eyelink 1000 eye tracking hardware (SR Research, Ottawa, Canada) in “desktop” configuration, tracking both eyes of the subjects at 500 Hz sampling frequency. This camera-based system utilizes infrared illumination to track the subject's pupil, and additionally a corneal reflection created by a separate light source. The reported raw gaze position is then computed internally by the system from the two individual measurements.

### Paradigm and Calibration

Each block of the experimental paradigm used for this study included a calibration phase and a fixation phase.

The calibration phase consisted of a series of fixation point grids with 5 rows and 5 columns, with a spacing of 5 deg visual angle between each row and column. The grids were centered on the screen and spanned a total area of 20×20 degree visual field. Each fixation point was designed as a small annulus (ca. 0.2 deg outer diameter) and only one point of the grid was shown at any given time. Subjects were asked to fixate the center of each fixation point as they appeared on the screen for at least one second; the paradigm switched to the next point when the subject pressed a button, but not before at least 800 ms had passed. Early key presses were ignored and required the subject to make a second attempt before the fixation point would move on. All points of the grid were cycled through in randomized order. The procedure was repeated 7 times per block, with a different background luminance on each repetition. Background luminance was varied systematically from black to white in 7 steps (0, 12.5, 25, 37.5, 50, 75 and 100% of the available screen luminance). The purpose of the different background intensities was to produce calibration data with both constricted and dilated pupil, as well as intermediate pupil sizes.

The fixation phase consisted of a period of ten seconds during which subjects were asked to maintain best possible fixation at a marker located at the center of the screen, and to avoid blinking. After 2 seconds of black background, during the following 2 seconds the background intensity then smoothly increased to white, where it remained for another 2 seconds before smoothly fading back to black, remaining there for the final 2 seconds. In each block, this procedure was repeated 3 times, allowing subjects to rest their eyes between repetitions. The fixation trials were used to measure drift characteristics over time, and to evaluate the effectiveness of the different compensation approaches.

Every recording session consisted of 3 blocks.

## Analysis and Results

39 subjects were tested (age: mean 23.4 (range: 19–35), 10M 29F). All subjects reported normal or corrected-to-normal eyesight at the time of the experiment and were payed for their participation. All data was subjected to manual scrutiny in order to detect unwanted blinks and eye movements, such as saccades during fixation.

### Calibration

For the purpose of this experiment, the built-in calibration mechanism of the eye tracker was not utilized, and raw, uncalibrated data was used to derive calibration solutions manually. No filtering or smoothing was applied. To match the raw eye tracker measurements to degrees of visual field, calibration solutions were computed separately for each recorded eye: For every point shown during the calibration phase (corresponding to one grid position), a 700 ms interval ending 300 ms before the subject's button press was cut, and the median of the eye position during this interval was computed. These medians were then averaged across the 3 repetitions of the calibration grid. Afterwards, separately for each background intensity, 2-dimensional polynomials of 3^rd^ order were fitted to project the averaged recorded fixation positions to the reference screen positions of the fixation markers. Each of these polynomials then formed one calibration solution specific to the respective background intensity, therefore representing a solution specific to the average pupil size induced by this same background intensity.

**Figure 1:**
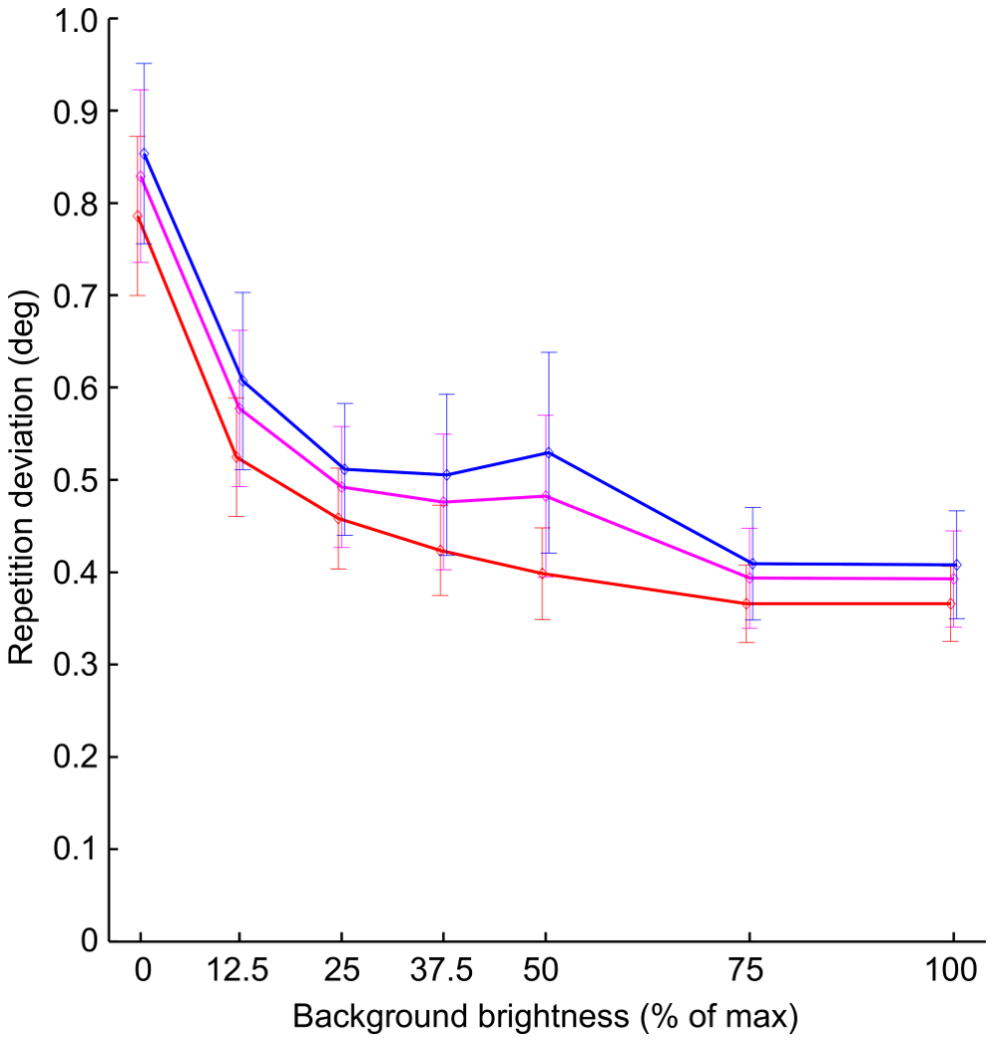
Mean deviation from calibration positions across repetitions, as measured during the calibration phase. Data averaged across subjects (mean and s.e.m.). The red curve represents data for the center quadrant only (−5 to +5 degrees in vertical and horizontal), the magenta curve represents data for the full calibration grid (−10 to +10 degrees in vertical and horizontal), the blue curve represents data for the outer region of the calibration grid (full calibration grid with the inner quadrant removed).

**Figure 2:**
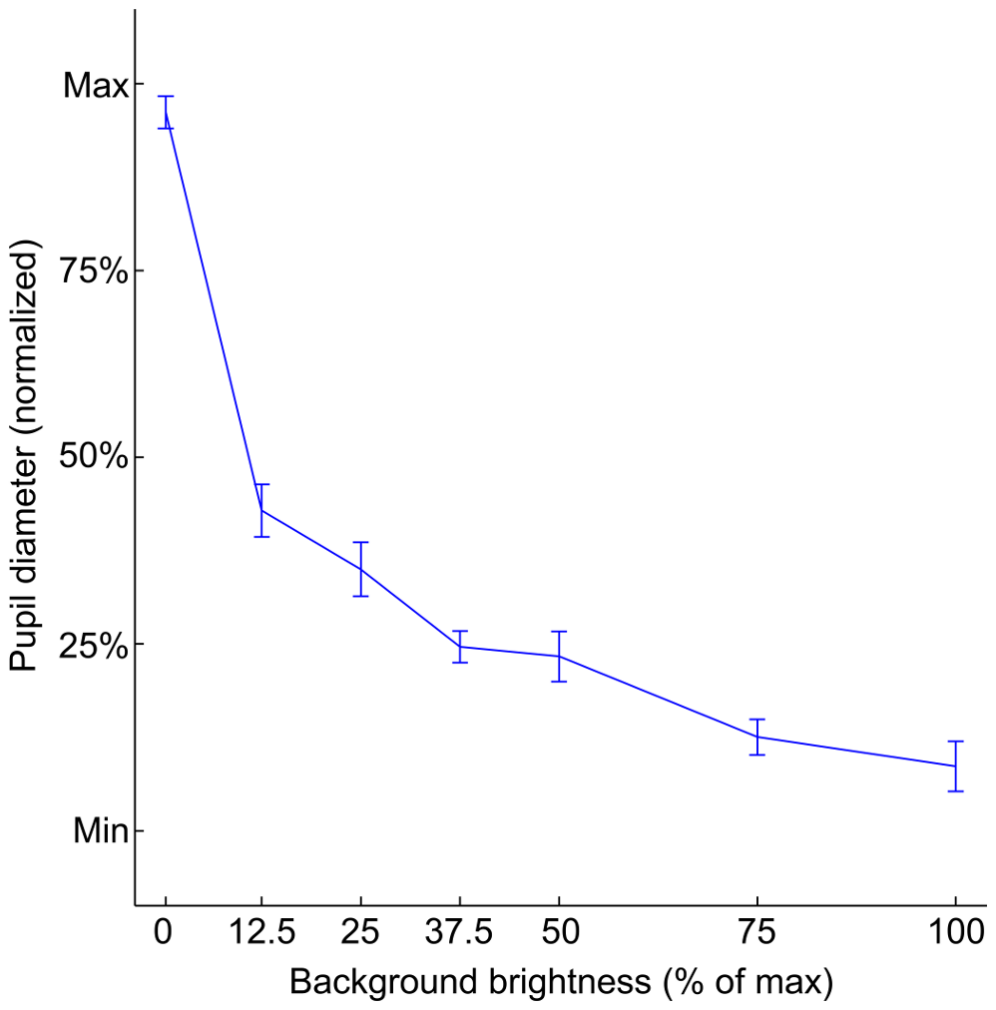
Average normalized pupil diameter vs. screen background brightness. Mean and s.e.m. across subjects.

Gaze calibration is never perfect. To determine the overall quality of the calibration solutions derived by the polynomial fitting, these solutions were applied to the fixation positions measured during the calibration phase. The mean deviation of the recorded fixations from their respective reference grid positions were then averaged first between the eyes of each subject, then across subjects. As the accuracy of camera-based eye trackers is usually best around the screen center (straight forward viewing), the repetitive accuracy was computed separately for the entire calibration grid, spanning 20×20 degrees (5×5 = 25 points), for the center quadrant, spanning 10×10 degrees (3×3 = 9 points), and for the outer region (the remainder after removal of the center quadrant, 16 points). In all three cases, the mean deviation was not constant and increased with decreasing background brightness and thus increasing pupil dilation (ANOVA on full field data, df = 6, F = 76.2, p<0.0001); the mean deviation was more than twice as large in the black background/dilated pupil condition compared to the white background, constricted pupil condition (0.83±0.09 and 0.39±0.05, degree±sem, full field data). Repetitive accuracy in the center quadrant was consistently better than in outer regions, although the difference was small, averaging 0.07degree (paired t-test, df = 6, p<0.001). The fixation accuracy results can be seen in Figure 1, the average pupil size for each background brightness can be seen in Figure 2. In general, the shapes of the graphs in Figures 1 and 2 correspond neatly to each other, indicating a strong correlation between repetitive accuracy and pupil size.

### Fixation/Drift Measurements

To compensate for any drifts that occurred outside of the trial measuring interval, all trials were centered by subtracting the median of an 800 ms interval beginning 1 s after trial start. To measure drift magnitude with a certain level of robustness against noise and small fixational eye movements, the median of the measured positional deviation was computed over a 2 s interval beginning at the moment when the screen background reached maximum intensity, as this was when subjects reached the minimum pupil size (maximal constriction). Each of the 3×3 trials performed by each subject was manually inspected; any trials with saccades or blinks during the critical intervals (centering and measuring) were discarded. Horizontal and vertical drift magnitude were determined separately, the overall drift magnitude was then computed by vector addition of the horizontal and vertical aspects and averaged over trials.

**Figure 3:**
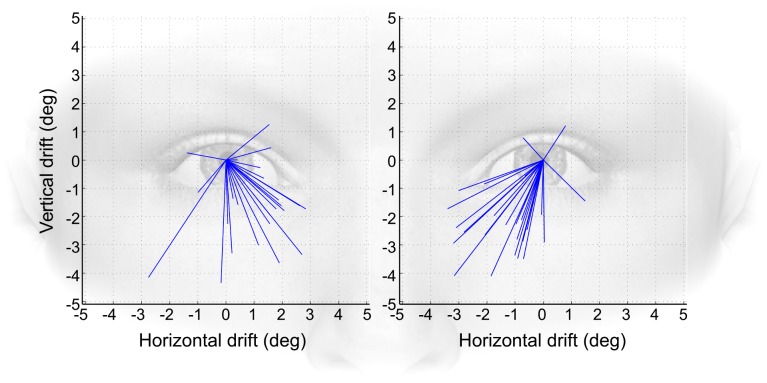
Drift distribution. Drifts represented as lines originating from a dilated pupil in direction of constriction. Dilated condition was centered at 0 degrees. Left panel displays data from right eyes, right panel displays data from left eyes. Symbolic face in the background to facilitate direction interpretation.

Measured drift magnitude exceeded the reports from previous studies. In many subjects, large differences in the drift magnitude were found between the left and the right eye; for this analysis, the left and right eyes of each subject were therefore treated as independent samples. Eyes were included in the analysis only when at least 4 valid trials were obtained, resulting in a total of 67 eyes. The maximum mean drift encountered in one subject measured 3.16 degrees in the horizontal and 4.09 degrees in the vertical, resulting in a total drift magnitude of 5.17 degrees (averaged from 9 valid trials). The smallest drift magnitude found was 0.34 degrees (7 valid trials). Across all samples, drift magnitude averaged 2.55 degrees. Measured in direction from dilation to constriction, most drift directions went in a downward-nasal direction (see Figure 3). When analyzing interocular difference within subjects, the average was 1.01 degrees, with a maximum of 3.01 and a minimum of 0.04 degrees.

### Drift Dynamics

To further examine drift dynamics, trials were rigorously cleaned such that there would be no blink, saccade or missing sample in the first 6 seconds of any surviving fixation trial. After this procedure, all sampled eyes with less than 4 remaining trials were ignored during further analysis. From 18 subjects, both eyes were found to have performed acceptably with another 8 subjects contributing data from one eye each, providing a total of 44 sample eyes (22 left, 22 right). All further analysis was based on these cleaned trials.

**Figure 4:**
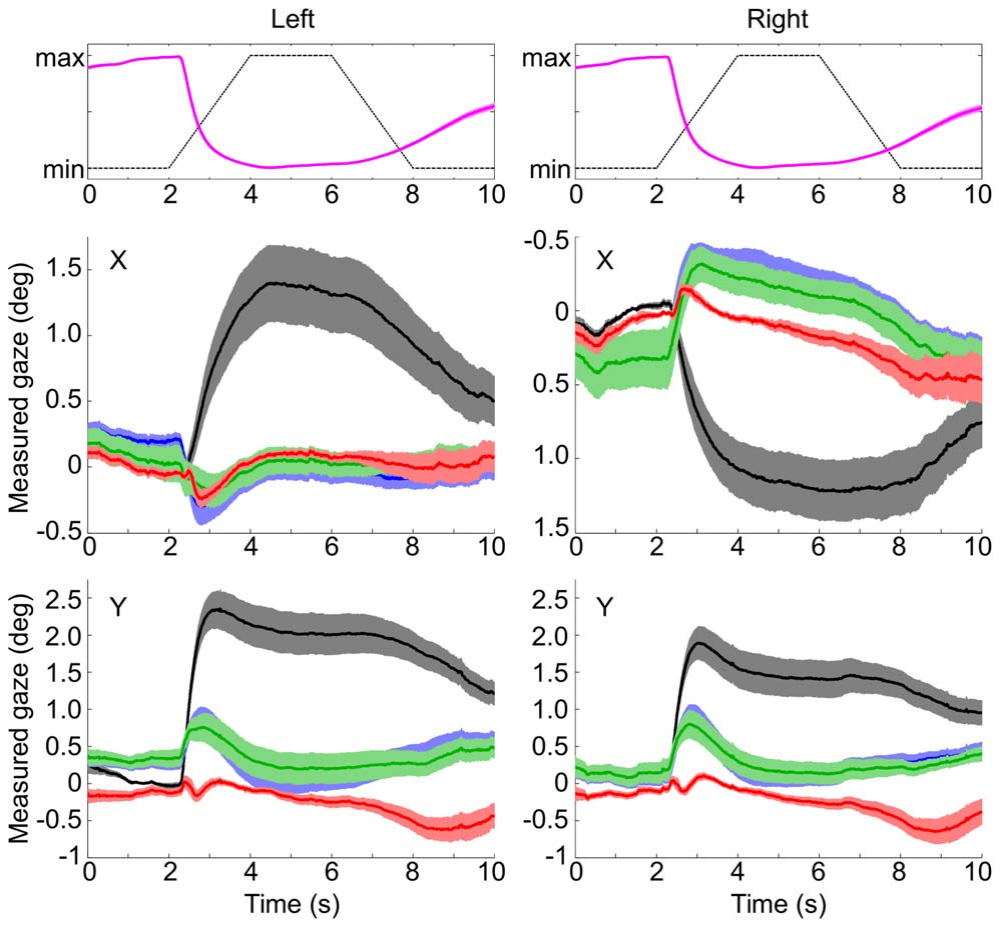
Drift and compensation time course. Top row: background brightness (black dashed) and normalized pupil response across subjects (magenta, mean and s.e.m.). Middle and bottom row: horizontal and vertical aspects of left and right eyes. Black/gray: uncorrected gaze recordings. Blue: 2-Point compensated. Green: 3-Point compensated. Red: LUT compensated. Mean and 1 s.e.m.

Uncompensated drift time courses for left and right eyes averaged across subjects can be seen from Figure 4.

The peak drift velocity was computed as the highest average velocity of any 50 ms interval between 2 s and 4 s after trial start (the interval of the fastest pupil constriction). The average peak velocity across sample was 8.4 deg/s (median: 8.0 deg/s), with a standard deviation of 3.5 deg/s and a range from 2.2 deg/s to 17.3 deg/s.

### Drift Compensation

While a patented method to compensate for pupil-size dependent shifts exists [12], most scientists will be unable to modify their eye tracking hardware to satisfy the needs of the patent's described methods. In the following, we will evaluate three attempts to compensate the pupil-size related drift in gaze measurement.

### Method 1: 2-Point

Although the pupil-size dependent shifts in measured gaze position we report here are in many cases complex, the first approximation of the observed drift is a simple linear function. With the help of two independent calibrations of the eye tracking system, one performed in a dilated-pupil (“dark”) condition and the other in a constricted-pupil (“bright”) condition, it should be possible to capture this linear component of the positional drift (see [11]). Based on two reference calibrations, a per-sample composite calibration may then be computed by weighting the “bright” and “dark” calibrations according to the current pupil diameter. If the pupil is as dilated as it was during the recording of the “dark” calibration, the weighting of the “dark” calibration should be 100% while the weighting of the “bright” calibration should be 0%. If the pupil diameter is half-way between the “bright” and “dark” conditions, both “bright” and “dark” calibrations should be weighted equally, and so on. This “2-Point” model results in the formula:

F1 (2-Point): 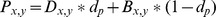


Where 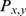 is the calibrated gaze position, compensated for pupil diameter; 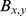 is the gaze data calibrated according to the “bright” condition, 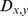 is the gaze data calibrated according to the “dark” condition, and 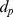 is the pupil diameter, scaled so that the average diameter during the “dark” condition is 1 and the average diameter during the “bright” condition is 0. The scaling is performed identically for both x and y aspects of the gaze data.

### Method 2: 3-Point

While the 2-Point compensation approach should be able to capture the linear component of the drift, better compensation might be achieved when taking a potential curvature of the underlying drift function into account. This is achieved by using an “intermediate” calibration in addition to the “dark” and “bright” ones used in the 2-Point approach. With 3 points of intercept, it is possible to fit of a quadratic function to the data, mapping pupil size to positional shift. For any given sample, the composite calibration is then computed from the “dark”, ”bright” and “intermediate” calibrations with the pupil diameter as a an index into the quadratic mapping function:

F2 (3-Point): 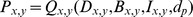


Where 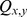 is the quadratic mapping function and 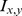 is the gaze data calibrated according to the “intermediate” condition.

To achieve best possible compensation, it is useful to adjust the “intermediate” calibration in such way that the average pupil size is as close as possible to the mid-point between “bright” and “dark” calibrations. In the case of the current study, the calibration with 12.5% background luminance was chosen for this purpose (see Figure 2).

### Method 3: Look Up Table (LUT)

The third compensation method is aimed at being able to compensate even highly irregular drift characteristics. By means of averaging over all valid trials, a sample-by-sample drift lookup table is constructed, mapping pupil size to drift magnitude. This approach has the advantage to be able to compensate for underlying drift functions of nearly arbitrary complexity, and it does only require a few fixation trials, rather than multiple calibrations with different luminance levels. A definite drawback however is that such a look-up table cannot account for a possible hysteresis between pupil constriction and pupil dilation. Here, we chose to generate the look-up table from the constriction phase of the fixation trials. In principle, the look up table may be formed by cutting the constriction phase of both the pupil and the gaze data, and then building a table that matches each sample in the pupil data with the drift magnitude at the same sample. Practically, measurement noise and small fluctuations in pupil size and gaze can cause this table to be ambiguous, as the same pupil size may occur more than once, paired with different drift values. In order to reduce noise and guarantee an injective (one-to-one) mapping, the pupil size curve was first averaged across trials, then approximated by means of fitting an exponential function to the averaged data of the given subject. The replacement of the sample-wise pupil data with a continuous, adapted function allows for continuous input values and eliminates the need for interpolation on the pupil-side of the algorithm. The measured drift values at each sample were then mapped to this function. As sampling frequency was 500 Hz, only very small amounts of drift are possible between two neighboring samples, allowing for a simple nearest-neighbor interpolation to fill any gaps in the resulting look-up table. The approach is described in the formula:

F3 (LUT): 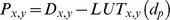


Where 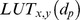 is the average drift magnitude, according to the look-up-table, indexed by the pupil diameter of the current sample.

### Compensation Results

**Figure 5:**
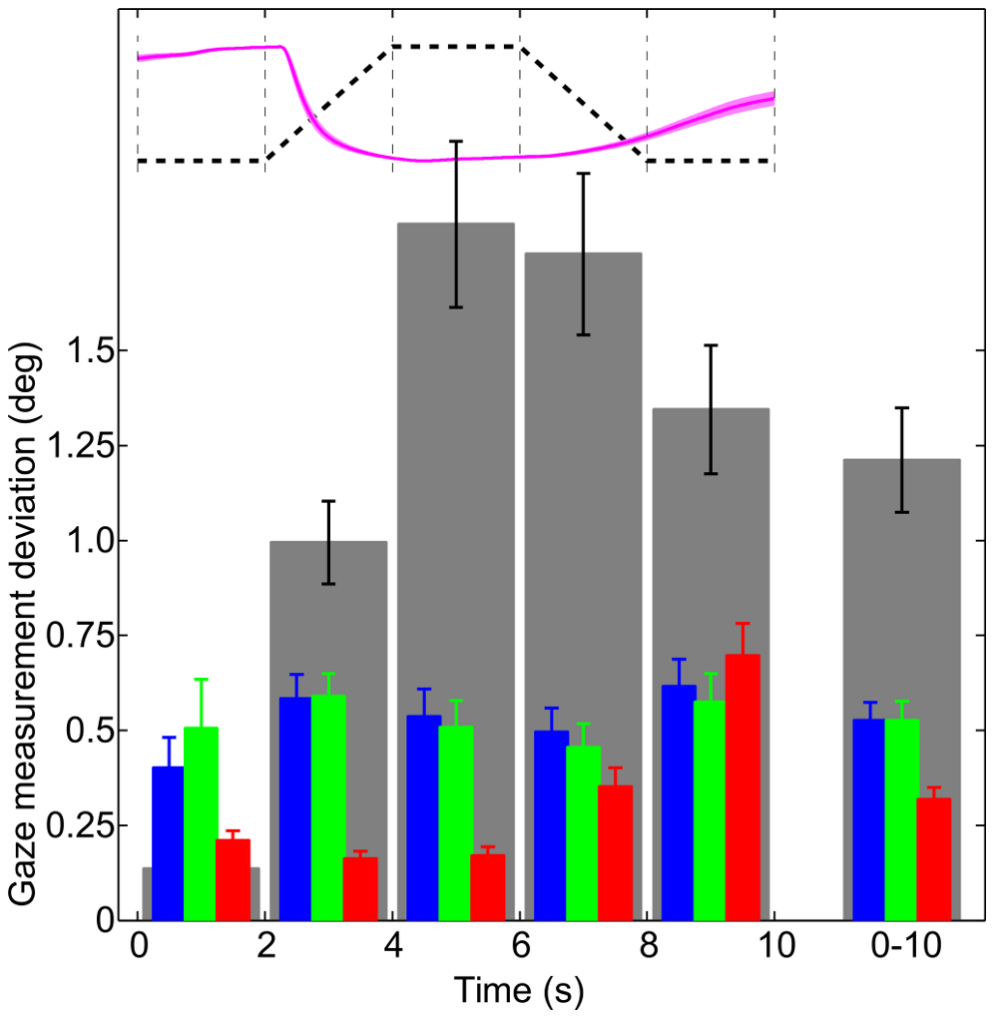
Drift compensation results. The top graph shows the profile of the background luminance time course (black dashed line) and the pupil size response (normalized, then averaged across subjects). The left group of bars shows the average rectified measurement deviation per 2-second interval on the same timescale (mean and 1 s.e.m.). The single right bar graph shows the average across the entire trial duration (10 s). Gray bars represent uncorrected measurements. Blue bars represent measurements compensated according to the 2-Point method. Green bars represent the 3-Point method. Red bars represent the LUT-Method.

All three attempted approaches succeeded in reducing drift magnitude, to varying amounts. Averaged time courses of compensated and raw data can be seen in Figure 4. Averaged across subjects and time, both the 2-Point and 3-Point methods reduced the amount of measured drift to 43% of the original value, while the LUT method was able to reduce the drift to only 26%. When dividing the fixation trials into the 5 basic periods (dark, luminance increase, bright, luminance decrease, and again dark, see Figure 5), it can be seen that of the three methods, the LUT method works best in the first four time intervals, while it achieves the worst compensation in the last time interval. This reveals the weakness of the look-up table approach: as the table was built from the constriction flank of the pupil size time course, the compensation is highly effective during the beginning of the trial, but less effective during the later, dilation-dominated phase. The 3-Point approach is actually just slightly superior to the 2-Point approach in all time intervals except the first.

## Discussion

The magnitude and direction of pupil-size induced measurement drifts were analyzed in a population of 39 subjects. Average drift magnitude was found to be 2.55°, with the average drift direction being downwards and towards the nasal plane (in direction of constriction). Notably, significant amounts of pupil-size induced drift were found in all subjects, without exception. Drift magnitude was variable both between subjects and between the left and right eyes of a given subject. Naturally, the pupil-size dependent drift is not the only drift ever to be found during fixation. Small drifts and microsaccades around the center of the fixation are to be expected and did occur in this study. However, all measures of drift magnitude and drift direction reported in this study are averages across multiple trials and are therefore dominated by pupil drift.

Eye tracker accuracies are reported by most eye tracker manufacturers, although the standards by which they are measured are not well defined [13]. For the optical system used in this work, an achievable accuracy of 0.25°–0.50° is specified by the manufacturer. This was approximately achieved in the data generated by our subjects, however only when the pupil was constricted to at least 50% relative to the individual minima and maxima. An average deviation of 2.55° together with a (short-term) apparent gaze velocity of 8.4°/s, solely due to a change in pupil size may add catastrophic amounts of noise to data collected during many common eye movement experiments.

In most psychophysical experiments, however, pupil size changes of this magnitude are not to be expected, as luminance changes are usually much smaller than the difference between a black screen and a full bright screen. Still, not every paradigm allows for perfect control of luminance (e.g. natural viewing outdoor conditions) and other causes for pupil size changes exist. Binocular vergence measurements may serve as an example, as they are particularly sensitive to symmetric measurement drifts. Pupil-size based artifacts might therefore easily be mistaken for vergence movements. With an assumed interocular distance of 65 mm and a viewing distance of 60 cm, the approximate version angle of each eye would be approx. 3.1°. As we found the average drift direction to be diagonal, vergence measurements will be affected only partially. As the overall drift magnitude in laboratory situations will certainly be smaller than the theoretical maximum, we may assume a horizontal drift of 0.25deg, resulting in a measured vergence distance of ca. 55 to 66 cm, or about 10% possible error. Measurement errors would be magnified at longer viewing distances. While luminance changes in controlled conditions may be small to negligible, outdoor eye tracking measurements may suffer from even larger changes in overall luminance, as the sun can of course be much brighter than any computer screen.

To improve gaze measurement accuracy, three methods of compensation were evaluated. The “2-Point” technique used 2 different calibrations, one on white and one on black background, and used the current pupil size as an index to interpolate between the resulting calibration solutions [11]. This method can easily be implemented independent of the utilized eye tracking hardware and is in principle real-time capable, but may easily be applied post-hoc and is therefore non-destructive. While the original, manufacturer-designed calibration procedure of the eye tracking system was not used in this study, the described methods would work equally well with pre-calibrated gaze data rather than raw values. The compensation may therefore be added as an optional second stage of calibration, and may be applied post-hoc at the researcher's discretion. It may be adjusted to the needs of specific setups by varying the number, density and position of the fixation dots. In particular, the compensation range may be approximated to the actual needs of the experiment, by using the maximum and minimum of the experiment design luminance instead of black and white. In doing so, the linear compensation of the 2-Point algorithm may best approximate the curved characteristics of the actual drift function, which would improve compensation effectiveness. The second proposed algorithm (“3-Point”) used an additional calibration at an intermediate luminance in order to improve on the linear compensation by means of a quadratic fit. The extra effort of a third calibration run seems ill invested however, as the gain in compensation effectiveness over the 2-Point algorithm was marginal at best. Finally, the third introduced algorithm used a look-up table built from individual drift traces, and achieved much better compensation effectiveness. However, the accuracy of the compensation depends on the direction of the pupil dynamics, and may therefore be useful only in specific circumstances.

In many eye tracking experiments, a compensation for pupil size changes may not be necessary. One possible indicator to determine whether one should be on the lookout for artefactual gaze dynamics would be time-locked changes in pupil size. Small gaze effects, for example in response to stimulus appearance, might be caused or affected by pupil dynamics and should warrant extra scrutiny to rule out measurement artifacts. If however there are no systematic changes in pupil size at the time of interest, then this kind of measurement artifact is not to be expected.

Generally, all three compensation methods may possibly introduce artificial gaze dynamics into the recorded data. When eye movement profiles, rather than positional accuracy, are in the focus of interest, then any gaze compensation methodology should be used with caution, or not at all. The 2-Point method would be the method of choice here if required, as it may only introduce linear dynamics, as opposed to the LUT-Method, which might introduce complex dynamics, depending on the individual subject. In some cases, it may be advisable to screen subjects for their potential drift magnitude prior to admitting them to a sensitive study. At last, with increasing pupil constriction, the magnitudes of both pupil size dynamics and the resulting measurement drift are diminished. A simple solution to avoiding pupil-size induced measurement inaccuracies may therefore be to run the experiment in a bright setting, e. g. with white screen background. This will cause pupil constriction, which will greatly reduce pupil dynamics - at least until the subject has adapted to the light settings.

In the past, the interaction between pupil size and measured gaze position has been very much neglected. For the future, some extra scrutiny, especially with regards to small effect sizes, would seem appropriate.
